# European H16N3 Gull Influenza Virus Attaches to the Human Respiratory Tract and Eye

**DOI:** 10.1371/journal.pone.0060757

**Published:** 2013-04-08

**Authors:** Cecilia Lindskog, Patrik Ellström, Björn Olsen, Fredrik Pontén, Debby van Riel, Vincent J. Munster, Daniel González-Acuña, Thijs Kuiken, Elsa Jourdain

**Affiliations:** 1 Department of Immunology, Genetics and Pathology, Science for Life Laboratory, Uppsala University, Uppsala, Sweden; 2 Department of Medical Sciences, Clinical Bacteriology, Uppsala University, Uppsala, Sweden; 3 Department of Medical Sciences, Infectious Diseases, Uppsala University, Uppsala, Sweden; 4 Erasmus Medical Centre, Rotterdam, The Netherlands; 5 Laboratory of Virology, Division of Intramural Research, National Institute of Allergy and Infectious Diseases, National Institutes of Health, Hamilton, Montana, United States of America; 6 Universidad Concepción, Chillán, Chile; 7 INRA UR346, St Genès Champanelle, France; Thomas Jefferson University, United States of America

## Abstract

We explored the attachment of an H16N3 influenza virus to human, mallard, and gull tissues using virus histochemistry applied to tissue microarrays and employing human and mallard viruses as references. Of the viruses tested, the H16N3 gull virus most readily attached to the human respiratory tract and eye. These results underscore the need to assess the potential for gull influenza viruses to replicate in human tissues and further investigate the role of gulls in influenza virus ecology.

## Introduction

Influenza A viruses have a wide range of host species, including humans, pigs, horses, dogs, wild mammals, and birds. However, the mechanisms that define this range remain unclear and require further study in order to understand the zoonotic potential of these viruses. In particular, more focus should be placed on wild waterbird species, which are considered to be the natural reservoir of influenza A viruses [1]. Viruses are classified into subtypes based on their specific combination of 2 antigenic surface proteins, hemagglutinin (H) and neuraminidase (N), of which there are 17 and 10 different types known, respectively [2]. Most H and N combinations have been found in wild waterbirds [1].

The roles played by different waterbird species in influenza virus transmission remain unclear. Important parameters such as the frequency and routes of interspecific transmission need to be clarified [1]. For instance, several recent field [3], [4] and experimental [5], [6] studies suggest that airborne transmission of low-pathogenic avian influenza (LPAI) viruses in ducks may be significant. Furthermore, more research is needed to identify species that may transmit viruses to domestic poultry or that harbor viruses with zoonotic potential. While most studies to date have focused on the Anseriformes, other groups of birds may also be important. Gulls, in particular, are often anthropophilic and carry specific virus subtypes characterized by H13 and H16 hemagglutinins [7], [8].

The successful transmission of an influenza virus is partially determined by the virus’ ability to attach to sialic acid residues on the surface of host cells. Avian viruses preferentially attach to alpha-2,3-linked sialic acids, whereas human viruses attach to alpha-2,6-linked sialic acids [9]. Virus histochemistry studies performed on tissues from humans and other mammals [10], as well as on those of wild [11] and domestic [12] birds, suggest that influenza virus receptor distribution is highly variable among species from the same taxonomic family or with similar feeding behaviors. The goal of the present study was to investigate the ability of an H16N3 influenza virus to attach to human tissues.

## Materials and Methods

We investigated the attachment of a gull virus (H16N3 A/black-headed gull/Sweden/2/99) to gull (*Leucophaeus pipixcan, Larus argentatus*), mallard (*Anas platyrhynchos*), and human tissues using a mallard virus (H6N1 A/mallard/Sweden/81/02) and a seasonal human influenza virus (H3N2 A/Netherlands/213/03) as references. The human virus was grown on Madin-Darby canine kidney (MDCK) cells whereas the gull and mallard isolates were obtained from cloacal swabs and subsequently passaged twice in embryonated chicken eggs. We chose to use an H16N3 virus because it is one of the most common subtypes identified in gulls [8]. This particular isolate was chosen because it was the first H16 virus to be isolated and the most representative of the isolates in our collection at the time of the experiment [13]. These specific H6N1 and H3N2 isolates were chosen as references because their attachment properties have been well characterized in various avian [11] and mammalian [10], [14], [15] species. We focused on tissues known to serve as replication sites for influenza viruses, namely those of the human respiratory tract and eye as well as those of the avian trachea and digestive tract. In addition, we checked for virus attachment to the human digestive tract.

Bird tissue sampling procedures were approved by the Swedish Environmental Protection Agency (permits number 412-6267-08NV/412-5977-08NV), the Swedish Board of Agriculture (permits number 74-08/43-09), the Chilean Agriculture Ministry (permit number 1-25-2008), and the Ethics Committee of the Veterinary University of Concepción (CE1-2006). All human tissues used were obtained from the Department of Pathology (Uppsala University Hospital, Uppsala, Sweden) as part of the Human Protein Atlas project (http://www.proteinatlas.org/) [16]. For each tissue type, we used samples from 4 human individuals and 3 fledgling birds of each avian species.

The tissues were formalin-fixed, paraffin-embedded, and included in 2 different tissue microarrays (TMAs, 98 cores in total corresponding to one core per original tissue block) as previously described [17], except for human eyes, which were studied separately on full tissue sections. Glass slides with 4 µm tissue sections were prepared, and virus attachment was detected by virus histochemistry [10]. Each tissue section was incubated with 50 hemagglutinating units of fluorescein-labeled influenza virus or phosphate buffer saline (which served as an omission control that checked for nonspecific staining). Tissues were counterstained with hematoxylin (Sigma-Aldrich, MO, USA), mounted with Vision Mount (Thermo Fisher Scientific, CA, USA), and scanned using Aperio ScanScope XT (Aperio Technologies, CA, USA) to generate high-resolution digital images. If influenza virus was attached to the tissues, the apical surface of the epithelium demonstrated granular to diffuse red staining. Two independent observers visually scored the fraction of positive cells of each investigated cell type. We scored each tissue once for each virus (one repetition per individual and tissue) and excluded tissue cores in which the investigated cell types were not visible.

## Results and Discussion

The results are summarized in Table 1 and detailed in Tables S1, S2, and S3. The attachment of human and mallard viruses to the human respiratory tract and mallard colon, respectively, was consistent with previously published results [10], [11], [14].

**Table 1:** Scores obtained for different tissues stained by virus histochemistry using human H3N2, mallard H6N1, and gull H16N3.

	Tissue	Number tested	H3N2	H6N1	H16N3
***Human***	Cornea	3, 3, 3	−	+	±
	Conjunctiva	2, 2, 2	−	+	+
	Nasopharynx	3, 3, 4	+	±	+
	Bronchus	4, 4, 4	+	±	+
	Lung	4, 4, 4	±	±	+
	Oral mucosa	2, 2, 2	−	±	−
	Salivary gland	4, 4, 4	−	−	±
	Esophagus	3, 3, 3	−	±	−
	Stomach	4, 4, 4	−	−	±
	Duodenum	4, 4, 4	−	−	−
	Small intestine	4, 4, 4	−	−	−
	Appendix	3, 3, 3	−	−	−
	Colon	4, 4, 4	−	−	−
	Rectum	4, 3, 3	−	−	−
***Anas platyhrynchos***	Trachea	1, 1, 2	−	+*	+
	Duodenum	3, 2, 3	−	+	−
	Ileum	3, 3, 3	−	+	−
	Ileocaecal junction	3, 3, 3	−	+	−
	Colon	2, 3, 3	−	+	−
***Larus argentatus***	Trachea	1, 1, 1	+*	−*	±*
	Duodenum	3, 3, 1	−	−	+
	Ileum	3, 3, 2	−	±	+
	Ileocaecal junction	2, 2, 1	−	±	+
	Colon	1, 1, 1	−	+	+
***Leucophaeus pipixcan***	Trachea	3, 3, 2	+	+	+
	Duodenum	3, 3, 3	−	+	+
	Ileum	2, 3, 2	−	+	+
	Ileocaecal junction	3, 3, 3	−	+	+
	Colon	2, 2, 2	−	+	+

The column “number tested” indicates the number of tissue cores tested for the human, mallard, and gull virus, respectively; the scores are as follows: − no attachment observed, ± attachment observed in at least one tissue core,+attachment to ≥50% of cells observed for at least one cell type in all tissue cores; the sign * indicates that very few cells were visible. Detailed scoring is provided in Tables S1, S2, and S3.

**Figure 1:**
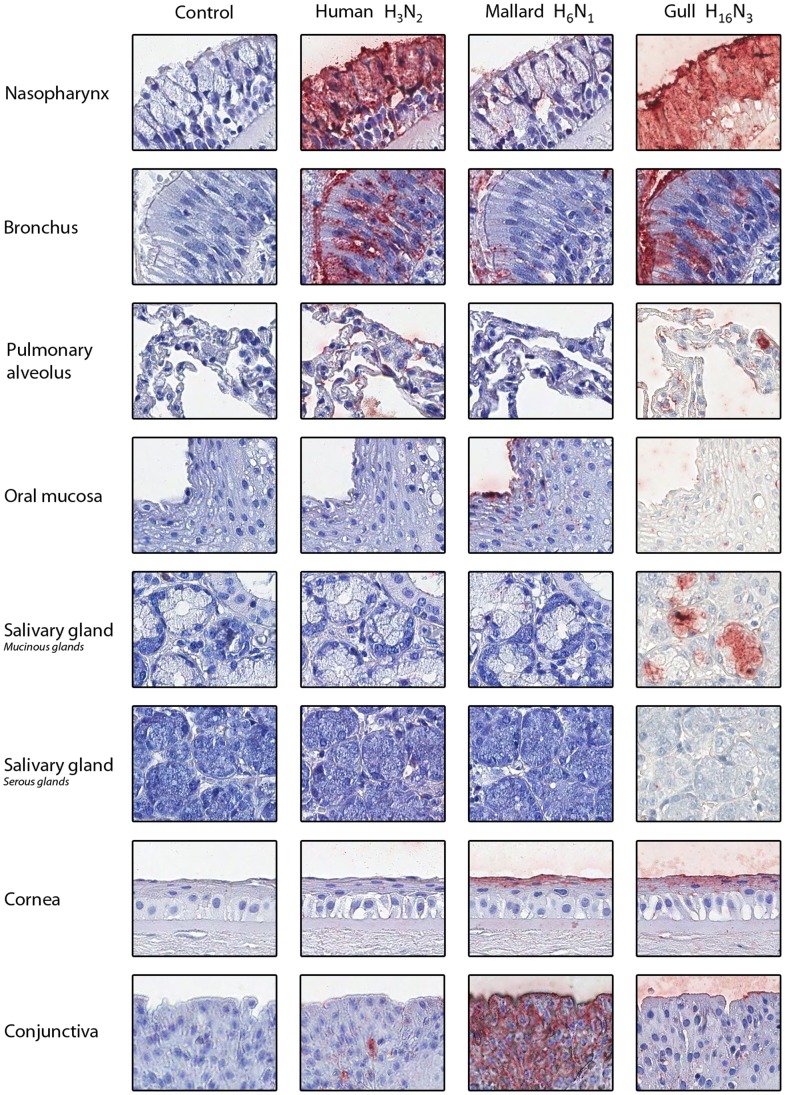
Attachment (in red) of human H3N2, mallard H6N1, and gull H16N3 influenza viruses to human tissues. The nuclei are counterstained with hematoxylin (blue). Control slides were incubated with phosphate buffer saline instead of fluorescein-labeled influenza viruses.

The human and gull viruses both attached to a majority of the ciliated and goblet cells in the human nasopharynx and bronchus (Figure 1), suggesting that this H16 gull virus might be more likely to replicate in the human respiratory tract than other avian influenza viruses tested thus far. Indeed, this result contrasts with those of previous studies showing that H5, H6, or H7 LPAI viruses attached to a moderate number of ciliated epithelial cells (always <50%) and no goblet cells within the human respiratory tract [10], [14]. In particular, the failure of H13 hemagglutinin to attach to human tracheal sections [18] suggests that the attachment abilities of H16 viruses may strongly differ from those of H13 viruses, although both are frequently isolated from gulls. The H16N3 virus attached to other human cell types as well, including alveolar macrophages and the epithelial cells of the cornea and conjunctiva. The mallard virus also attached to the human eye, a result that had already been observed for LPAI viruses that induce conjunctivitis in humans [19]. Conversely, although attachment of the mallard virus to the oral mucosa and esophagus suggests that receptor molecules are expressed by these tissues, it seems unlikely that they serve as virus entry routes because their epithelium is pluristratified. Only the H16 gull influenza virus attached to the salivary mucous glands (*i.e*., one tissue core). Avian influenza viruses have been previously observed to attach to glandular cells, namely those of the submucosal glands of the upper respiratory tract, trachea, and bronchus [10], [14]. Both saliva and mucus are potential barriers for influenza viruses because multiple components of human saliva and mucus have been shown to inhibit influenza virus infection [20], [21]. However, further studies are necessary to assess whether this H16 gull influenza virus can be neutralized by human saliva or mucus.

**Figure 2:**
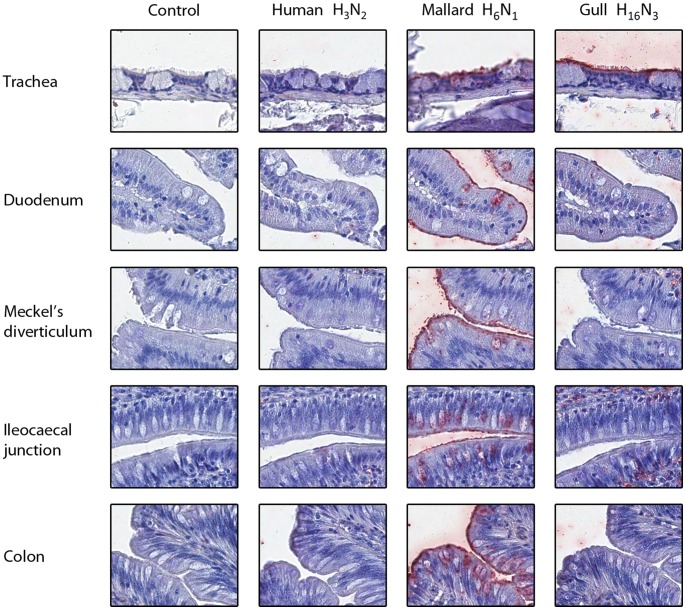
Attachment (in red) of human H3N2, mallard H6N1, and gull H16N3 influenza viruses to mallard tissues. The nuclei are counterstained with hematoxylin (blue). Control slides were incubated with phosphate buffer saline instead of fluorescein-labeled influenza viruses.

**Figure 3:**
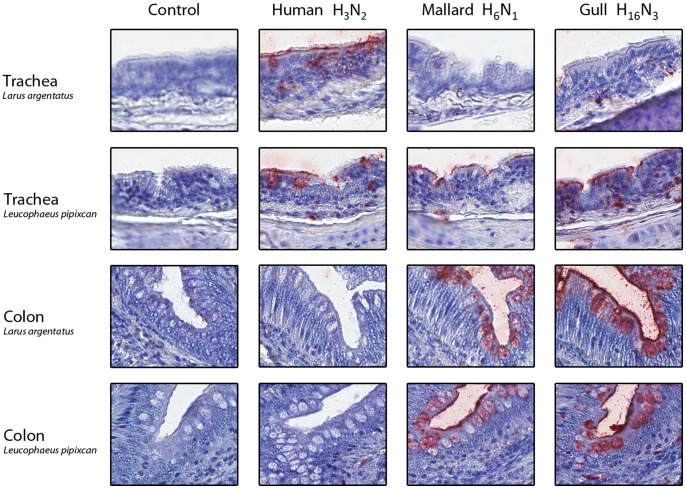
Attachment (in red) of human H3N2, mallard H6N1, and gull H16N3 influenza viruses to gull tissues. The nuclei are counterstained with hematoxylin (blue). Control slides were incubated with phosphate buffer saline instead of fluorescein-labeled influenza viruses.

As expected, the human virus did not attach to any of the mallard tissues tested. However, the gull virus attached to the mallard trachea (Figure 2), and the mallard virus attached to the mallard trachea and intestinal tissues. This result suggests that avian influenza virus replication is not restricted to the colon in ducks, but rather occurs along the entire digestive tract, which is consistent with immunohistochemistry observations of naturally infected mallards [22] and experimentally infected domestic ducks [23]. We observed differential attachment of duck and gull viruses. This observation concurs with past work that suggests these viruses differ in their attachment properties [24], even if both H13 and H16 viruses have occasionally been isolated from ducks [7], [8]. The human virus attached to the trachea of both gull species (Figure 3), suggesting that the first step necessary for human to gull transmission of influenza A virus can occur. The mallard and gull viruses attached to both *L. pipixcan* and *L. argentatus* tissues, although attachment showed some tissue-dependent variation. This result is consistent with field data reporting that H6 viruses are regularly isolated from gulls in both Eurasia and America [8], [25].

### Conclusions

Attachment to target cells is the first step of influenza virus infection, regardless of the virus’ ability to subsequently establish a productive infection in tissues. Although experimental infection is needed to confirm that infection is possible in a particular species, comparing virus attachment patterns in different hosts may indicate which species are likely to share similar viruses and which tissues are likely to serve as virus attachment sites. In this study, we successfully used TMAs to screen and compare the attachment patterns of viruses isolated from humans, mallards, and gulls, which indicates that this technique could be used to screen larger sets of influenza viruses in the future. The results obtained for this H16N3 virus suggest that H16 influenza viruses, which are largely gull-specific, may present a different cell tropism in mallards and humans than do other avian influenza viruses. In particular, the H16N3 virus we used attached more readily to the human respiratory tract than did the other avian influenza viruses and it also attached to the human cornea and conjunctiva. Because some genetic variation exists within the H16 sequences currently known [8], [13] and viruses of the same subtype may vary in their attachment patterns [26], further studies investigating the attachment potential of other H16 influenza viruses are needed to determine whether the observed attachment properties can be generalized to all H16 influenza viruses. We also showed that human seasonal flu virus attached to the trachea of both gull species investigated, which is the first necessary step in human to gull virus transmission. Given that gulls may be highly anthropophilic [27], these results raise concerns about the potential transmission of influenza viruses between humans and gulls. The likelihood of such transmission events should be further elucidated in detailed and functional studies that explore the additional factors that contribute to successful cross-species transmission, of which attachment is only the first step.

## Supporting Information

Table S1 Attachment of human H3N2 virus in human, mallard and gull tissues. The column “n” indicates the number of individuals tested; the scores are as follows: – no attachment observed, ± attachment observed in at least one tissue core,+attachment to ≥50% of cells observed for at least one cell type in all tissue cores; the sign * indicates that only few cells were visible.
(DOCX)

Table S2 Attachment of mallard H6N1 virus in human, mallard and gull tissues. The column “n” indicates the number of individuals tested; the scores are as follows: – no attachment observed, ± attachment observed in at least one tissue core,+attachment to ≥50% of cells observed for at least one cell type in all tissue cores; the sign * indicates that only few cells were visible.
(DOCX)

Table S3 Attachment of gull H16N3 virus in human, mallard and gull tissues. The column “n” indicates the number of individuals tested; the scores are as follows: – no attachment observed, ± attachment observed in at least one tissue core,+attachment to ≥50% of cells observed for at least one cell type in all tissue cores; the sign * indicates that only few cells were visible.
(DOCX)
