# Supplementary material for: European H16N3 Gull Influenza Virus Attaches to the Human Respiratory Tract and Eye
Source: PLoS One. 2013 Apr 8;8(4):e60757. doi: 10.1371/journal.pone.0060757 (PMC3620227; doi:10.1371/journal.pone.0060757)
Supplement: Table S2 — Attachment of mallard H6N1 virus in human, mallard and gull tissues. The column “n” indicates the number of individuals tested; the scores are as follows: – no attachment observed, ± attachment observed in at least one tissue core,+attachment to ≥50% of cells observed for at least one cell type in all tissue cores; the sign * indicates that only few cells were visible. (DOCX) [file pone.0060757.s002.docx]

| **Mallard H6N1 virus** | **Tissue** | **n** | **Score** | **Comments** |
| --- | --- | --- | --- | --- |
| ***Human*** | Cornea | 3 | + | attachment to >75% surface epithelial cells |
|  | Conjunctiva | 2 | + | attachment to >75% surface epithelial cells |
|  | Nasopharynx | 3 | ± | low (<25%, n=2) or no (n=1) attachment to ciliated cells; low (<25%, n=1) or no (n=1) attachment to goblet cells |
|  | Bronchus | 4 | ± | no (n=3) or low (<25%, n=1) attachment to ciliated cells; attachment to <50% goblet cells (n=2) |
|  | Pulmonary alveolus | 4 | ± | no attachment to alveolar cells (n=4); no (n=3) or very low (<10%, n=1) attachment to macrophages |
|  | Oral mucosa | 2 | ± | attachment to >75% (n=1) or <25% (n=1) epithelial cells |
|  | Salivary gland | 4 | - | no attachment to serous glands (n=4) and mucinous glands (n=1) |
|  | Esophagus | 3 | ± | attachment to >50% (n=2) or >25% (n=1) epithelial cells |
|  | Stomach | 4 | - | no attachment to surface epithelial cells of lower and upper stomach |
|  | Duodenum | 4 | - | no attachment to epithelial and goblet cells |
|  | Small intestine | 4 | - | no attachment to epithelial and goblet cells |
|  | Appendix | 3 | - | no attachment to epithelial and goblet cells |
|  | Colon | 4 | - | no attachment to epithelial and goblet cells |
|  | Rectum | 3 | - | no attachment to epithelial and goblet cells |
| ***Anas platyhrynchos*** | Trachea | 1 | +* | attachment to >75% ciliated and goblet cells |
|  | Duodenum | 2 | + | attachment to >75% epithelial and goblet cells; crypts not visible |
|  | Ileum | 3 | + | attachment to >75% (n=2) or >50% (n=1) epithelial and goblet cells, and to >75% (n=1) or >50% (n=1) crypt cells |
|  | Ileocaecal junction | 3 | + | attachment to >75% epithelial and goblet cells; attachment to >50% crypt cells (n=1) |
|  | Colon | 3 | + | attachment to >75% epithelial and goblet cells; attachment to >75% crypt cells (n=1) |
| ***Larus argentatus*** | Trachea | 1 | -* | no attachment to ciliated and goblet cells |
|  | Duodenum | 3 | - | no attachment to epithelial and goblet cells; crypts not visible |
|  | Ileum | 3 | ± | no attachment to epithelial cells (n=3); no (n=2) or very low (<10%, n=1) attachment to goblet cells; no attachment to crypt cells (n=1) |
|  | Ileocaecal junction | 2 | ± | no attachment to epithelial cells (n=2); very low (<10%, n=1) attachment to goblet cells; crypts not visible |
|  | Colon | 1 | + | very low (<10%) attachment to epithelial cells; attachment to >25% goblet cells, and to >75% crypt cells |
| ***Leucophaeus pipixcan*** | Trachea | 3 | + | attachment to >75% ciliated cells (n=3) and to >50% goblet cells (n=2) |
|  | Duodenum | 3 | + | attachment to >75% epithelial cells (n=2); to >50% (n=1) or <25% (n=1) goblet cells, and to >75% crypt cells (n=2) |
|  | Ileum | 3 | + | attachment to >75% epithelial cells (n=3); to >75% (n=1) or >50% (n=1) goblet cells, and to >75% crypt cells (n=2) |
|  | Ileocaecal junction | 3 | + | attachment to >75% epithelial, goblet and crypt cells |
|  | Colon | 2 | + | attachment to >75% epithelial, goblet and crypt cells |
