# Supplementary material for: European H16N3 Gull Influenza Virus Attaches to the Human Respiratory Tract and Eye
Source: PLoS One. 2013 Apr 8;8(4):e60757. doi: 10.1371/journal.pone.0060757 (PMC3620227; doi:10.1371/journal.pone.0060757)
Supplement: Table S3 — Attachment of gull H16N3 virus in human, mallard and gull tissues. The column “n” indicates the number of individuals tested; the scores are as follows: – no attachment observed, ± attachment observed in at least one tissue core,+attachment to ≥50% of cells observed for at least one cell type in all tissue cores; the sign * indicates that only few cells were visible. (DOCX) [file pone.0060757.s003.docx]

| **Gull H16N3 virus** | **Tissue** | **n** | **Score** | **Details** |
| --- | --- | --- | --- | --- |
| ***Human*** | Cornea | 3 | ± | attachment to >75% (n=1), <50% (n=1) or <25% (n=1) surface epithelial cells |
|  | Conjunctiva | 2 | + | attachment to >75% (n=1) or >50% (n=1) surface epithelial cells |
|  | Nasopharynx | 4 | + | attachment to >75% ciliated (n=4) and goblet (n=2) cells |
|  | Bronchus | 4 | + | attachment to >75% ciliated (n=4) and goblet (n=2) cells |
|  | Pulmonary alveolus | 4 | + | low (<25%) attachment to alveolar cells; attachment to >75% macrophages |
|  | Oral mucosa | 2 | - | no attachment to epithelial cells |
|  | Salivary gland | 4 | ± | no attachment to serous glands (n=4); attachment to >75% mucinous glands (n=1) |
|  | Esophagus | 3 | - | no attachment to epithelial cells |
|  | Stomach | 4 | ± | no (n=3) or very low (<10%, n=1) attachment to surface epithelial cells of lower and upper stomach |
|  | Duodenum | 4 | - | no attachment to epithelial and goblet cells |
|  | Small intestine | 4 | - | no attachment to epithelial and goblet cells |
|  | Appendix | 3 | - | no attachment to epithelial and goblet cells |
|  | Colon | 4 | - | no attachment to epithelial and goblet cells |
|  | Rectum | 3 | - | no attachment to epithelial and goblet cells |
| ***Anas platyhrynchos*** | Trachea | 2 | + | attachment to >75% ciliated and goblet cells |
|  | Duodenum | 3 | - | no attachment to epithelial and goblet cells; crypts not visible |
|  | Ileum | 3 | - | no attachment to epithelial and goblet cells; no attachment to crypt cells |
|  | Ileocaecal junction | 3 | - | no attachment to epithelial and goblet cells; no attachment to crypt cells |
|  | Colon | 3 | - | no attachment to epithelial and goblet cells; no attachment to crypt cells |
| ***Larus argentatus*** | Trachea | 1 | ±* | no attachment to ciliated cells; very low (<10%) attachment to goblet cells |
|  | Duodenum | 1 | + | attachment to>75% epithelial and goblet cells; crypts not visible |
|  | Ileum | 2 | + | attachment to>75% epithelial cells; attachment to >75% (n=1) or >25% (n=1) goblet cells; crypts not visible |
|  | Ileocaecal junction | 1 | + | attachment to>75% epithelial and goblet cells; crypts not visible |
|  | Colon | 1 | + | attachment to>75% epithelial, goblet and crypt cells |
| ***Leucophaeus pipixcan*** | Trachea | 2 | + | attachment to >50% ciliated cells; very low (<10%) attachment to goblet cells |
|  | Duodenum | 3 | + | attachment to >75% epithelial cells (n=2); low (<25%, n=2) attachment to goblet cells; attachment to >75% crypt cells (n=2) |
|  | Ileum | 2 | + | attachment to >75% epithelial cells, <50% goblet cells, and to >75% crypt cells (n=1) |
|  | Ileocaecal junction | 3 | + | attachment to >75% epithelial cells, >50% goblet cells, and to >75% crypt cells |
|  | Colon | 2 | + | attachment to >75% epithelial, goblet and crypt cells |
